# 1-(4-Chloro­benzyl­ideneamino)pyridinum iodide

**DOI:** 10.1107/S1600536808043729

**Published:** 2009-01-08

**Authors:** Yong-Tao Cui, Jian-Qiang Wang, Chun-Xiang Ji, Hai-Bo Wang, Guo Cheng

**Affiliations:** a College of Science, Nanjing University of Technolgy, Xinmofan Road No. 5 Nanjing, Nanjing 210009, People’s Republic of China

## Abstract

In the title compound, C_12_H_10_ClN_2_^+^·I^−^, the aromatic rings are oriented at a dihedral angle of 54.55 (3)°. In the crystal structure, inter­molecular C—H⋯I and C—H⋯Cl hydrogen bonds link the mol­ecules.

## Related literature

For background, see: Okamoto *et al.* (1967 ▶). For bond-length data, see: Allen *et al.* (1987 ▶).
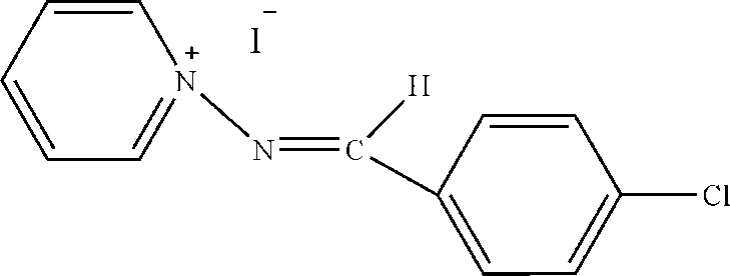


## Experimental

### 

#### Crystal data

C_12_H_10_ClN_2_^+^·I^−^
*M* *_r_* = 344.57
Triclinic, 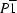

*a* = 6.5105 (14) Å
*b* = 7.1748 (15) Å
*c* = 14.223 (3) Å
α = 76.893 (3)°
β = 79.183 (3)°
γ = 80.753 (3)°
*V* = 630.7 (2) Å^3^
*Z* = 2
Mo *K*α radiation
μ = 2.72 mm^−1^
*T* = 291 (2) K
0.10 × 0.10 × 0.08 mm

#### Data collection

Enraf–Nonius CAD-4 diffractometer
Absorption correction: ψ scan (North *et al.*, 1968 ▶) *T*_min_ = 0.772, *T*_max_ = 0.812
3220 measured reflections
2205 independent reflections
1892 reflections with *I* > 2σ(*I*)
*R*_int_ = 0.063
3 standard reflections frequency: 120 min intensity decay: none

#### Refinement

*R*[*F* ^2^ > 2σ(*F* ^2^)] = 0.027
*wR*(*F* ^2^) = 0.093
*S* = 1.05
2205 reflections
145 parameters
H-atom parameters constrained
Δρ_max_ = 0.53 e Å^−3^
Δρ_min_ = −0.63 e Å^−3^

### 

Data collection: *CAD-4 Software* (Enraf–Nonius, 1989 ▶); cell refinement: *CAD-4 Software*; data reduction: *XCAD4* (Harms & Wocadlo, 1995 ▶); program(s) used to solve structure: *SHELXS97* (Sheldrick, 2008 ▶); program(s) used to refine structure: *SHELXL97* (Sheldrick, 2008 ▶); molecular graphics: *ORTEP-3 for Windows* (Farrugia, 1997 ▶); software used to prepare material for publication: *SHELXL97*.

## Supplementary Material

Crystal structure: contains datablocks global, I. DOI: 10.1107/S1600536808043729/hk2600sup1.cif

Structure factors: contains datablocks I. DOI: 10.1107/S1600536808043729/hk2600Isup2.hkl

Additional supplementary materials:  crystallographic information; 3D view; checkCIF report

## Figures and Tables

**Table 1:** Hydrogen-bond geometry (Å, °)

*D*—H⋯*A*	*D*—H	H⋯*A*	*D*⋯*A*	*D*—H⋯*A*
C1—H1⋯I1	0.93	3.04	3.857 (5)	147
C5—H5⋯Cl1^i^	0.93	2.79	3.691 (6)	162

Symmetry code: (i) 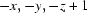.

**Fig. 1.**
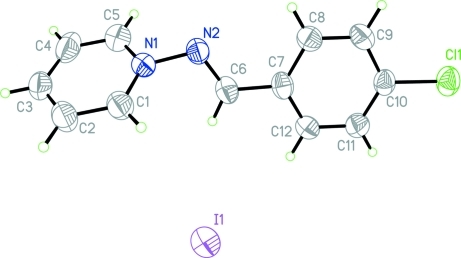
The molecular structure of the title molecule, with the atom-numbering scheme. Displacement ellipsoids are drawn at the 50% probability level.

**Fig. 2.**
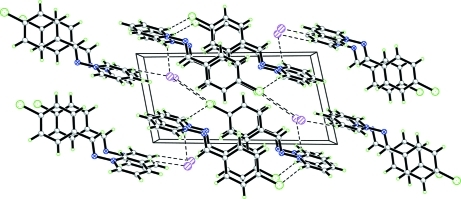
A partial packing diagram of the title compound. Hydrogen bonds are shown as dashed lines.

## supplementary crystallographic information

### Comment

Some derivatives of 1-aminopyidium iodide are important chemical materials. We report herein the crystal structure of the title compound.

In the molecule of the title compound (Fig. 1), the bond lengths (Allen *et al.*,  1987) and angles are within normal ranges. Rings A (N1/C1-C5) and B (C7-C12) are, of course, planar, and they are oriented at a dihedral angle of 54.55 (3)°.

In the crystal structure, intramolecular C-H···I and intermolecular C-H···Cl hydrogen bonds (Table 1) link the molecules (Fig. 2), in which they may be effective in the stabilization of the structure. The π-π contacts between the pyridine rings and the benzene rings, Cg1—Cg1^i^ and Cg2—Cg2^ii^ [symmetry codes: (i) 1 - x, -y, -z; (ii) 1 - x, -y, 1 - z, where Cg1 and Cg2 are centroids of the rings A (N1/C1-C5) and B (C7-C12) , respectively] may further stabilize the structure, with centroid-centroid distances of 4.130 (3) Å and 4.056 (3) Å.

### Experimental

For the preparation of the title compound, 1-aminopyridinium iodide (22.2 g, 0.10 mol) was dissolved in ethanol (20 ml), 4-methylbenzaldehyde (12.4 g, 0.1 mol) was added with stirring, and then the mixture was heated at reflux for 5 h. Upon cooling to room temperature, a precipitate formed, which was collected by filtration and washed with cold ethanol (2 X 10 ml) to obtain a yellow solid (yield; 24.0 g, 70%). Crystals suitable for X-ray analysis were obtained by slow evaporation of an ethanol solution.

### Refinement

H atoms were positioned geometrically, with C-H = 0.93 Å for aromatic and methine H and constrained to ride on their parent atoms, with U_iso_(H) = 1.2U_eq_(C).

### Crystal data

**Table:**

C_12_H_10_ClN_2_^+^·I^−^	*Z* = 2
*M**_r_* = 344.57	*F*(000) = 332
Triclinic, *P*1	*D*_x_ = 1.814 Mg m^−^^3^
Hall symbol: -P 1	Mo *K*α radiation, λ = 0.71073 Å
*a* = 6.5105 (14) Å	Cell parameters from 25 reflections
*b* = 7.1748 (15) Å	θ = 2.1–25.3°
*c* = 14.223 (3) Å	µ = 2.72 mm^−^^1^
α = 76.893 (3)°	*T* = 291 K
β = 79.183 (3)°	Block, yellow
γ = 80.753 (3)°	0.10 × 0.10 × 0.08 mm
*V* = 630.7 (2) Å^3^	

### Data collection

**Table:**

Enraf–Nonius CAD-4 diffractometer	1892 reflections with *I* > 2σ(*I*)
Radiation source: fine-focus sealed tube	*R*_int_ = 0.063
graphite	θ_max_ = 25.0°, θ_min_ = 2.9°
ω/2θ scans	*h* = −7→7
Absorption correction: ψ scan (North *et al.*, 1968)	*k* = −8→8
*T*_min_ = 0.772, *T*_max_ = 0.812	*l* = −15→16
3220 measured reflections	3 standard reflections every 120 min
2205 independent reflections	intensity decay: none

### Refinement

**Table:**

Refinement on *F*^2^	Primary atom site location: structure-invariant direct methods
Least-squares matrix: full	Secondary atom site location: difference Fourier map
*R*[*F*^2^ > 2σ(*F*^2^)] = 0.027	Hydrogen site location: inferred from neighbouring sites
*wR*(*F*^2^) = 0.093	H-atom parameters constrained
*S* = 1.05	*w* = 1/[σ^2^(*F*_o_^2^) + (0.0479*P*)^2^] where *P* = (*F*_o_^2^ + 2*F*_c_^2^)/3
2205 reflections	(Δ/σ)_max_ < 0.001
145 parameters	Δρ_max_ = 0.53 e Å^−^^3^
0 restraints	Δρ_min_ = −0.63 e Å^−^^3^

### Special details

**Table:**

Geometry. All e.s.d.'s (except the e.s.d. in the dihedral angle between two l.s. planes) are estimated using the full covariance matrix. The cell e.s.d.'s are taken into account individually in the estimation of e.s.d.'s in distances, angles and torsion angles; correlations between e.s.d.'s in cell parameters are only used when they are defined by crystal symmetry. An approximate (isotropic) treatment of cell e.s.d.'s is used for estimating e.s.d.'s involving l.s. planes.
Refinement. Refinement of *F*^2^ against ALL reflections. The weighted *R*-factor *wR* and goodness of fit *S* are based on *F*^2^, conventional *R*-factors *R* are based on *F*, with *F* set to zero for negative *F*^2^. The threshold expression of *F*^2^ > σ(*F*^2^) is used only for calculating *R*-factors(gt) *etc*. and is not relevant to the choice of reflections for refinement. *R*-factors based on *F*^2^ are statistically about twice as large as those based on *F*, and *R*- factors based on ALL data will be even larger.

### Fractional atomic coordinates and isotropic or equivalent isotropic displacement parameters (Å^2^)

**Table:**

	*x*	*y*	*z*	*U*_iso_*/*U*_eq_	
I1	0.91966 (5)	0.25912 (5)	0.16564 (2)	0.05410 (17)	
Cl1	0.1793 (2)	0.4285 (2)	0.64323 (10)	0.0562 (3)	
N1	0.4087 (6)	−0.1676 (5)	0.2056 (3)	0.0410 (9)	
N2	0.3499 (6)	−0.1179 (6)	0.3000 (3)	0.0434 (9)	
C1	0.6038 (8)	−0.1437 (7)	0.1537 (4)	0.0495 (12)	
H1	0.7003	−0.0912	0.1775	0.059*	
C2	0.6541 (10)	−0.1996 (7)	0.0648 (4)	0.0608 (15)	
H2	0.7863	−0.1852	0.0274	0.073*	
C3	0.5076 (10)	−0.2770 (7)	0.0313 (4)	0.0585 (14)	
H3	0.5404	−0.3131	−0.0291	0.070*	
C4	0.3107 (11)	−0.3012 (8)	0.0878 (4)	0.0654 (16)	
H4	0.2111	−0.3533	0.0658	0.079*	
C5	0.2669 (9)	−0.2469 (7)	0.1763 (4)	0.0562 (13)	
H5	0.1380	−0.2654	0.2160	0.067*	
C6	0.3740 (8)	0.0561 (7)	0.2976 (3)	0.0451 (11)	
H6	0.4240	0.1326	0.2382	0.054*	
C7	0.3254 (7)	0.1391 (7)	0.3855 (3)	0.0415 (11)	
C8	0.2488 (7)	0.0346 (7)	0.4776 (3)	0.0442 (11)	
H8	0.2289	−0.0937	0.4851	0.053*	
C9	0.2026 (8)	0.1216 (7)	0.5574 (3)	0.0474 (12)	
H9	0.1510	0.0536	0.6189	0.057*	
C10	0.2355 (7)	0.3145 (7)	0.5436 (3)	0.0424 (11)	
C11	0.3049 (8)	0.4181 (7)	0.4539 (3)	0.0479 (12)	
H11	0.3202	0.5476	0.4461	0.058*	
C12	0.3526 (8)	0.3297 (7)	0.3745 (3)	0.0460 (11)	
H12	0.4033	0.3992	0.3132	0.055*	

### Atomic displacement parameters (Å^2^)

**Table:**

	*U*^11^	*U*^22^	*U*^33^	*U*^12^	*U*^13^	*U*^23^
I1	0.0580 (3)	0.0587 (3)	0.0457 (2)	−0.01153 (17)	−0.00263 (16)	−0.01233 (16)
Cl1	0.0519 (8)	0.0723 (9)	0.0497 (7)	−0.0091 (6)	−0.0010 (6)	−0.0280 (6)
N1	0.041 (2)	0.043 (2)	0.039 (2)	−0.0075 (18)	−0.0030 (18)	−0.0090 (17)
N2	0.045 (2)	0.048 (2)	0.038 (2)	−0.0064 (18)	−0.0026 (17)	−0.0132 (17)
C1	0.055 (3)	0.048 (3)	0.043 (3)	−0.004 (2)	−0.004 (2)	−0.009 (2)
C2	0.076 (4)	0.050 (3)	0.046 (3)	0.001 (3)	0.009 (3)	−0.010 (2)
C3	0.083 (4)	0.047 (3)	0.044 (3)	−0.009 (3)	0.000 (3)	−0.016 (2)
C4	0.094 (5)	0.050 (3)	0.061 (4)	−0.006 (3)	−0.023 (3)	−0.021 (3)
C5	0.060 (4)	0.055 (3)	0.055 (3)	−0.009 (3)	−0.008 (3)	−0.014 (3)
C6	0.050 (3)	0.049 (3)	0.036 (3)	0.001 (2)	−0.008 (2)	−0.010 (2)
C7	0.037 (3)	0.050 (3)	0.038 (3)	0.003 (2)	−0.008 (2)	−0.014 (2)
C8	0.041 (3)	0.046 (3)	0.043 (3)	−0.004 (2)	−0.003 (2)	−0.009 (2)
C9	0.047 (3)	0.056 (3)	0.036 (3)	−0.007 (2)	0.000 (2)	−0.008 (2)
C10	0.034 (3)	0.056 (3)	0.041 (3)	−0.006 (2)	−0.006 (2)	−0.017 (2)
C11	0.055 (3)	0.044 (3)	0.046 (3)	−0.008 (2)	−0.009 (2)	−0.009 (2)
C12	0.052 (3)	0.047 (3)	0.037 (3)	−0.010 (2)	−0.006 (2)	−0.003 (2)

### Geometric parameters (Å, °)

**Table:**

Cl1—C10	1.746 (5)	C5—H5	0.9300
N1—C5	1.331 (7)	C6—C7	1.465 (6)
N1—C1	1.359 (6)	C6—H6	0.9300
N1—N2	1.434 (5)	C7—C12	1.377 (6)
N2—C6	1.275 (6)	C7—C8	1.399 (6)
C1—C2	1.379 (7)	C8—C9	1.378 (6)
C1—H1	0.9300	C8—H8	0.9300
C2—C3	1.380 (8)	C9—C10	1.399 (7)
C2—H2	0.9300	C9—H9	0.9300
C3—C4	1.393 (9)	C10—C11	1.358 (7)
C3—H3	0.9300	C11—C12	1.380 (6)
C4—C5	1.367 (7)	C11—H11	0.9300
C4—H4	0.9300	C12—H12	0.9300
			
C5—N1—C1	123.4 (4)	N2—C6—H6	119.0
C5—N1—N2	116.0 (4)	C7—C6—H6	119.0
C1—N1—N2	120.4 (4)	C12—C7—C8	119.8 (4)
C6—N2—N1	112.2 (4)	C12—C7—C6	117.4 (4)
N1—C1—C2	118.0 (5)	C8—C7—C6	122.8 (4)
N1—C1—H1	121.0	C9—C8—C7	120.1 (4)
C2—C1—H1	121.0	C9—C8—H8	119.9
C1—C2—C3	119.7 (5)	C7—C8—H8	119.9
C1—C2—H2	120.1	C8—C9—C10	118.4 (4)
C3—C2—H2	120.1	C8—C9—H9	120.8
C2—C3—C4	120.2 (5)	C10—C9—H9	120.8
C2—C3—H3	119.9	C11—C10—C9	121.8 (4)
C4—C3—H3	119.9	C11—C10—Cl1	118.7 (4)
C5—C4—C3	118.6 (5)	C9—C10—Cl1	119.5 (4)
C5—C4—H4	120.7	C10—C11—C12	119.5 (4)
C3—C4—H4	120.7	C10—C11—H11	120.2
N1—C5—C4	120.0 (5)	C12—C11—H11	120.2
N1—C5—H5	120.0	C7—C12—C11	120.4 (4)
C4—C5—H5	120.0	C7—C12—H12	119.8
N2—C6—C7	122.0 (4)	C11—C12—H12	119.8

### Hydrogen-bond geometry (Å, °)

**Table:**

*D*—H···*A*	*D*—H	H···*A*	*D*···*A*	*D*—H···*A*
C1—H1···I1	0.93	3.04	3.857 (5)	147
C5—H5···Cl1^i^	0.93	2.79	3.691 (6)	162

Symmetry codes: (i) −*x*, −*y*, −*z*+1.
